# P050: Containment of methicillin resistant staphylococcus aureus (MRSA) outbreak in a neonatal intensive care unit (NICU)

**DOI:** 10.1186/2047-2994-2-S1-P50

**Published:** 2013-06-20

**Authors:** O Eluk, Y Shachor-Meyouhas, Y Geffen, S Blazer, I stein, I Kassis

**Affiliations:** 1 Pediatric Infectious Diseases and Control Unit, Rambam Health Care Campus, Haifa, Israel; 2 Neonatal Intensive Care Unit, Rambam Health Care Campus, Haifa, Israel

## Introduction

MRSA infections in NICU are associated with significant morbidity and mortality. Early containment of outbreaks is crucial. Trials comparing different methods of screening and decolonization are lacking

## Objectives

To describe an epidemiologic and molecular investigation of MRSA outbreak in NICU

## Methods

Our NICU is a 25 bedslevel III unit. The main space has 9 beds for critically ill neonates. Two rooms serve as intermediate care (8 beds each). Almost 540 neonates are admitted a year. The index case was an 8 days old term baby. MRSA was isolated from his infected eye. Infection control team set an immediate investigation and emergency policy including: cohorting of MRSA^+^ cases, strict isolation and separate nursing team. All infants were screened for MRSA from nares, throat, axilla, groin, rectum, twice weekly, until one month after the last case discharged. Health care workers (HCW) and parents of positive cases were screened, re-educated for infection control measures and updated daily. NICU was closed until all colonized infants were detected and isolated. Visiting was restricted. MRSA isolates were collected for molecular testing.

## Results

Four colonized neonates were immediately identified by first screening. One patient was discharged and the rest were isolated in a separate room. Another infant was identified 20 days later. The last MRSA+ neonate was discharged 3 month later. HCW and families screening was negative. MRSA was isolated from five infants by nasal and rectal swabs; one was detected from axilla only. Two MRSA+ patients already known in the PediatricIntensive Care Unit (PICU) located near the NICU were suspected to be the source. All NICU Isolates were identical by PFGE. The two PICU isolates were different from each other and from NICU isolates. NICU and one PICU isolates were defined as ST-5 strain by MLST. One PICU isolate was ST-627. All isolates were PVL negative and SCCmec type IV. No further cases were detected. No cases of MRSA infection occurred during the outbreak period.

## Conclusion

Outbreaks of MRSA are hazardous in the NICU. Strict infection control policy and active screening may abort outbreaks.

## Disclosure of interest

None declared

